# Food allergies in developing and emerging economies: need for comprehensive data on prevalence rates

**DOI:** 10.1186/2045-7022-2-25

**Published:** 2012-12-20

**Authors:** Joyce Irene Boye

**Affiliations:** 1 Food Research and Development Centre, Agriculture and Agri-Food Canada, 3600 Casavant Boul West, St Hyacinthe, Quebec J2S 8E3, Canada

**Keywords:** Food allergy, Food hypersensitivity, Nutrition, Developing countries

## Abstract

Although much is known today about the prevalence of food allergy in the developed world, there are serious knowledge gaps about the prevalence rates of food allergy in developing countries. Food allergy affects up to 6% of children and 4% of adults. Symptoms include urticaria, gastrointestinal distress, failure to thrive, anaphylaxis and even death. There are over 170 foods known to provoke allergic reactions. Of these, the most common foods responsible for inducing 90% of reported allergic reactions are peanuts, milk, eggs, wheat, nuts (e.g., hazelnuts, walnuts, almonds, cashews, pecans, etc.), soybeans, fish, crustaceans and shellfish. Current assumptions are that prevalence rates are lower in developing countries and emerging economies such as China, Brazil and India which raises questions about potential health impacts should the assumptions not be supported by evidence. As the health and social burden of food allergy can be significant, national and international efforts focusing on food security, food safety, food quality and dietary diversity need to pay special attention to the role of food allergy in order to avoid marginalization of sub-populations in the community. More importantly, as the major food sources used in international food aid programs are frequently priority allergens (e.g., peanut, milk, eggs, soybean, fish, wheat), and due to the similarities between food allergy and some malnutrition symptoms, it will be increasingly important to understand and assess the interplay between food allergy and nutrition in order to protect and identify appropriate sources of foods for sensitized sub-populations especially in economically disadvantaged countries and communities.

## Introduction

Food allergy is the consequence of maladaptive immune responses to common and otherwise innocuous food antigens [1]. The most common allergenic foods are cow’s milk and dairy products, hen’s egg, peanuts, nuts, gluten containing cereals (e.g., wheat, rye, barley), sesame, soybeans, mustard, fish, crustaceans and shellfish [2-6]. These specific food substances are frequently referred to as “priority food allergens” as they account for over 90% of all food allergic reactions. Other less common allergenic foods include legumes, and some fruits and fruit juices (e.g., kiwifruit, apple, grape) and vegetables (e.g., celeriac, carrots, onions).

Rising prevalence of food allergy and intolerance in the developed world has attracted much attention in the last two decades. Consequently, there is a growing body of knowledge about the prevalence, health and social impacts of food allergy in many developed countries. Work done by the Food Allergy Research and Resource Program (FARRP) and other institutions in the United States of America (USA) have allowed allergy prevalence rates in the country to be estimated at 3.5-4.0% [7,8]. A nationwide study in the USA specifically focusing on peanut and tree nut allergy reported 1% overall prevalence rate of allergy to these foods [9]. Various international initiatives such as the InformAll database and EuroPrevall, a European Union funded project which focused on the prevalence, cost and basis of food allergy in Europe have provided very useful data on prevalence rates across Europe [10,11]. Estimates of prevalence based on food challenge studies ranged from 0% to 3% for milk, 0% to 1.7% for egg and 1% to 10.8% for any food. In Canada, Ben-Shoshan *et al.*[12] reported probable prevalence rates of 0.9%, 1.1%, 0.5%, 1.4% and 0.1% to peanut, tree nut, fish, shellfish (specific type not specified) and sesame. Similar studies have been conducted in Australia and Japan [13]. Together, such studies have helped to raise awareness of food allergies which has resulted in national and international legislation and recommendation for the identification of priority allergens when present in foods.

Unfortunately, in many countries in the developing world and in some emerging economies (e.g., China, India and Brazil) there continues to be a paucity of information regarding the prevalence and incidence of food allergy and other food hypersensitivities. Current assumptions are that prevalence rates are lower but this raises questions about potential health impacts should the assumptions not be supported by evidence. As the health and social burden of food allergy can be significant, national and international efforts focusing on food security, food quality and dietary diversity need to pay special attention to the role of food allergy in order to avoid marginalization of sub-populations in the community. This is also of importance for non-governmental organizations and international organizations involved in food aid programs as the major sources of foods used in emergency and food aid programs are considered as priority allergens (e.g., peanut, soybean, wheat, milk, eggs).

The objective of the present paper is to review the emerging body of evidence on the prevalence of food allergy in developing and emerging economies and compare the major foods implicated with those identified in the developed world. The review is intended to be succinct and comprehensive but is not exhaustive. The approach used was as follows: an extensive literature review was done using Scopus and Medline with the key words “food allergy AND Asia”, “food allergy AND Middle east”, “food allergy AND Africa” and “food allergy AND South America” covering the years 1972 to 2012. A hand search was also done on Science Direct with similar key words to identify studies with relevant titles. A few reports were also found through a Google search. Table 1 provides the number of relevant reports found and the region of study. A list of the most common allergenic foods in some of these different geographic areas is provided in Table 2. Whereas a comparison of the incidence and prevalence of food allergies in developed, emerging and developing economies would have been useful, a systematic comparison could not be undertaken due to a dearth of information from various geographic regions and differences in methodologies and approaches used in the limited studies found. Of particular note is that most available prevalence rates found for many of the developing and emerging countries are based on questionnaires and self-reported allergies which frequently overestimate true food allergy. This review thus uses a narrative approach rather than a meta-analysis and concludes by highlighting the need for more comprehensive studies. With increasing international trade and growing demands for food to feed an anticipated 9 billion by 2050, collaborative international efforts will be required in food safety and nutrition especially in regards to food allergy and food intolerance.

**Table 1:** Number of reports found on food allergy for different geographic regions

**Geographic region**	**Number of papers found**	**Specific countries in geographic region**
Asia	21	China, Hong Kong, India, Indonesia, Malaysia, Philippines, Singapore, South Korea, Taiwan, Thailand
Middle East	8	Iran, Israel, Saudi Arabia
Africa	27	Botswana, Egypt, Ghana, Morocco, Mozambique, Nigeria, South Africa, Togo, Zimbabwe
South America	15	Argentina, Brazil, Chile, Mexico

**Table 2:** Major allergenic foods in various geographic regions

**Geographic region**	**Country**	**Most common allergens reported**	**References**
Asia	China	Shellfish, egg, peanut, beef, cow’s milk, tree nuts	[15]
		Egg, cow’s milk, peanut, soy, wheat,	[13]
	Hong Kong	Milk, egg, fish, wheat, soy, peanut	[13]
	Thailand	Shellfish, peanut, soy, rice, egg, milk	[13]
	Philippines	Milk, shellfish, egg, fish, wheat, soy	[13]
	Taiwan	Egg, milk, peanut, soy, shellfish, wheat	[13]
	Indonesia	Peanut, shellfish, fish, egg, milk, rice	[13]
	Malaysia	Milk, soy, egg, fish, rice	[13]
	Singapore	Shellfish, milk, egg, wheat, peanut, soy	[13]
		Egg, shellfish, peanut, fish, cow’s milk, sesame	[15]
	Korea	Egg, milk, fish, pork, seafood (6–12 yr)	[21]
		Seafood, milk, peach, egg, fish (12–15 yr)	
Middle East	Saudi Arabia	Peanut, egg, cow’s milk, wheat, banana, fish	[30]
	Israel	Egg, milk, sesame	[32]
Africa	Mozambique	Seafood, meat, fruits/vegetables	[42]
	Zimbabwe	Apple, tomato, soy, crab, peanut	[35]
	Morocco	Egg, peanut, wheat	[45]
	Ghana	Peanut, pineapple	[41]
	South Africa	Wheat, peanut, fish, soy, egg, milk	[36]
	Egypt	Peanut, fish, egg, cow’s milk, sesame, banana	[44]
South America	Mexico	Dairy, egg, fish, shrimp, beans, soy	[51]
		Fish, milk, seafood, soy, orange	[55]
		Wheat, dairy, shellfish, pork, egg	[59]
	Brazil	Fish, egg, milk, wheat, peanut, soy, corn	[27]

### Prevalence of food allergies in Asia

Recent studies describing the pattern of anaphylaxis and the role of food triggers show that food is an important cause of severe allergic reactions in Asia [14]. A study by Leung *et al.*[15] of 3677 Chinese pre-school children aged 2–7 yr living in Hong Kong showed prevalence rates of parent-reported adverse food reaction (AFR) and parent-reported, doctor-diagnosed AFR to be 8.1% and 4.6%, respectively. The six leading causes of AFR were shellfish (15.8%), egg (9.1%), peanut (8.1%), beef (6.4%), cow’s milk (5.7%), and tree nuts (5.0%). Although the reasons are not clear, children born in mainland China had less parent-reported AFR (4.0%) than children born and raised in Hong Kong (6.7%). AFR was concluded to be a common atopic disorder in Hong Kong pre-school children, with prevalence rates comparable to that found in Caucasians.

Hill *et al.*[13] found similar allergy prevalence rates in young children in Australia and several countries in Asia (Hong Kong, China, Taiwan, Indonesia, Philippines, Malaysia, Singapore, Japan, Thailand). The major difference was that culprit allergenic foods for Asia were different. Prevalence rates of food hypersensitivity in Australian infants and children were 3.2% for egg, 2% for cow’s milk, 1.9% for peanut, 1.2% for tree nuts and sesame, 0.2% for wheat, 0.1% for soy and 0.1% for fish. In Asia, hypersensitivity to shellfish and fish was more common than for nuts, peanut and wheat. This was particularly so when fish was part of the staple infant diet. With the exception of a few reported cases, rice hypersensitivity was rare in both Australia and the Asian countries considered.

Prevalence of peanut, tree nut, and shellfish allergy in schoolchildren in Singapore and the Philippines was studied by Shek *et al.*[16]. Peanut and tree nut allergy was comparatively lower in Asian children compared to expatriate children (i.e., children living and attending expatriate/international schools in Singapore but who were born outside Asia), and a higher dominance of shellfish allergy was noted. Shellfish allergy prevalence in local Singapore was 1.2% for children aged 4 to 6 years and 5.2% for 14 to 16 year olds. In the Philippines, the prevalence rate was 5.1% for 14–16 year olds (no data available for 4 to 6 year olds). Convincing peanut and tree nut allergy were similar in local school children living in Singapore (4–6 years, 0.6%, 0.3%; 14–16 years, 0.5%, 0.3%, respectively) and the Philippines (14–16 years, 0.4%, 0.3%, respectively). Prevalence rates for expatriate children for both peanut and tree nuts ranged between 1.1 -1.3% and 0.6 – 1% for shellfish allergy.

Chiang *et al.*[17] characterized food protein sensitization patterns in 227 children in Singapore who presented to an allergy clinic over 3 years with symptomatic allergic disease and at least one specific food allergen sensitization documented on skin prick testing. Egg, shellfish, peanut, fish, cow’s milk, sesame, wheat and soy were the major culprits. Ninety (40%) of the positive skin tests were positive to egg, 87 (39%) to shellfish, 62 (27.3%) to peanut, 30 (13.2%) to fish, 27 (11.8%) to cow’s milk, 21 (9.3%) to sesame, 13 (3.7%) to wheat and 8 (3.2%) to soy. Children having multiple food hypersensitivities and a family history of atopic dermatitis were found to be more likely to present with peanut allergy. Interestingly, shellfish sensitization was determined to be higher in children with allergic rhinitis who were sensitized to cockroaches (tropomyosin is a major allergen found in many shellfish and also insects like cockroaches). In a follow up study with 31 children with a positive skin prick test to peanut, 87.1% had IgE specific to both Ara h 1 and Ara h 2 and 54.8% to Ara h 3 [17]. Major allergens that induce IgE-responses in peanut-sensitive patients are Ara h 1, Ara h 2 and Ara h 3, 4 [18]. The authors, thus, demonstrated that Asian children with peanut sensitization have clinically similar presentations and respond to the same major allergenic proteins as their Western counterparts.

In addition to the allergens mentioned above, Goh *et al.*[19] reported the Chinese delicacy bird’s nest soup to be the most common food allergen source in Singapore. The soup is made from the nest of swiftlets (tropical birds mostly found in South-east Asia). Unlike other birds, swiftlets secrete a gummy saliva which they use in building their nest. The saliva hardens when secreted to form the nest and there is hardly any plant material (i.e., twigs, leaves) found in the nest. Bird’s nest soup is a delicacy believed to increase longevity. This belief informs dietary habits in certain regions in Asia resulting in exposure of infants and children to bird’s nest at a very early age. Bird’s nest soup provides a good example of how dietary habits contribute to food allergy.

A retrospective study by Yang *et al.*[20] of 138 patients with anaphylaxis, including inpatients, outpatients, and emergency department visitors, in the Seoul National University Hospital in Korea covering the period January 1, 2000, through July 31, 2006 found food and food-dependent exercise-induced to be responsible for 21.3% and 13.2% anaphylaxis events, respectively. Buckwheat was the leading cause of food anaphylaxis. Earlier studies by Oh *et al.*[21] of prevalence of food allergy in Korean school children 6–15 year olds found rates ranging from 4.7 to 5.1% (children with diagnoses) and 5.7 to 8.6% (children with symptoms in the last 12 months of the study). Depending on the age group, the most common food allergens were egg, cow’s milk, fish, seafood (specific type not indicated), pork and peach.

In addition to the priority allergens, sensitization to some pulse legumes has been reported in Asia. Kumari *et al.*[22] investigated blackgram sensitization in asthma and rhinitis patients in India. Of 816 patients, 16 patients (~2%) were skin prick test (SPT) positive and 14 (1.7%) showed elevated specific IgE to blackgram. Four of the 14 patients reacted in oral challenge studies. Although not confirmed clinically, a high cross-reactivity has been reported for different legumes (e.g., soybeans, peanuts, pulses) [23,24]. Blackgram, for example, shares allergenicity with lentil and limabean [22]. The clinical relevance of such cross-reactivities needs to be carefully considered. Hegde *et al.*[25] also reported an allergic reaction to pomegranate in India.

### Prevalence of food allergies in the Middle East

Very few studies have been conducted on food allergy prevalence rates in the Middle East. Food was the fourth trigger of allergic reaction in a study conducted in Mashhad in Northeast Iran [26]. Prevalence of all allergic disorders in the city was 27.5% (data includes all allergic disorders and is not restricted to food allergies). In another study, 35.9 % of asthmatic children in Semnan (Iran) showed sensitization to at least one of the principal allergenic foods (wheat, rice, peanut, egg, soya and cow’s milk) [27].

GadElRab [28] in a study of 217 patients suffering from asthma, rhinitis and urticaria in Riyadh, Saudi Arabia found 17.5% to have specific IgE antibodies to various foods. The most common sensitivities were to peanut (23%), egg (15%) and cow’s milk (13%). Further studies with 100 adult asthmatic patients found up to 58% reacting to at least one of 24 allergens tested with the most common allergens being peanut (11%), egg white (3%), milk (3%), wheat (3%), banana (3%) and fish (2%) [29]. Aba-Alkhail and El-Gamal [30] similarly reported a 29% prevalence rate of clinical sensitivity to food in a study of 1341 asthmatic patients in Jeddah, Saudi Arabia. Another study of patients attending an outpatient allergy clinic in Riyadh, Saudi Arabia, found 13% to be sensitized to date fruits [31].

In Israel, prevalence of clinically revelant IgE-mediated food allergic reaction in 9070 infants and young children was 1.2%, with egg, cow’s milk and sesame being the most common food allergens identified [32]. Another study linked increased dispensing rate of adrenaline auto-injectors (an increase by 76% between 1997 and 2004) to potential increased rates of anaphylactic reactions, increased awareness of the risk of anaphylaxis or both [33]. The authors further observed that the lack of reported cases of anaphylactic death may, amongst others, be partly explained by under-diagnosis and under-reporting and recommended further studies to determine prevalence rates of anaphylactic reactions.

### Prevalence of food allergies in Africa

Data on food allergy prevalence rates in Africa is limited. Approximately 10% of 14,000 patients of all ages referred to the only specialist allergy clinic in Harare, Zimbabwe, in the 5-year period from September 1997 to September 2002, were reportedly diagnosed with food allergies [34]. Westritschnig *et al.*[35] conducted a study of 50 allergic patients in Zimbabwe for the presence of IgE antibodies to 20 food allergen extracts. Apple (24%), tomato (24%), soy (22%), crab (22%) and peanut (20%) were the most frequently detected food allergens.

Using skin prick tests, Levin *et al*. [36] reported 5% prevalence rate of food allergy in a cross-sectional study of 211 urban high school black children of Xhosa ethnicity in South Africa. Foods causing most allergies were egg white (3.3%), peanut (1.9%) and milk (1.9%). Wheat, soy and fish have been reported as common allergens in other studies [37]. In one study, mothers in South Africa consuming more peanut during pregnancy were found to have higher probability of having a child with peanut allergy [38]. Significant associations between peanut sensitization and soy and codfish sensitivity were reported. In regards to seafood, an earlier study found prawns (46.7%), crayfish (43.8%), abalone (35.2%), black mussels (33.3%), oyster (23.8%), snails (16.2%), shrimp (13.3%), crab (12.4%) and squid (11.4%) to be some of the most common species causing adverse food reactions in South Africa [39]. Bony fish species most commonly causing reactions were hake (24.8%), yellowtail (21.9%), salmon (15.2%), mackerel (15.2%), kingklip (13.3%) and snoek (10.5%). In another similar study of the sera of 80 seafood allergic South African patients, the highest sensitization was found to crustaceans (50%) followed by molluscs (30%) and a variety of fish species (20%) [40].

In Ghana, a study of food allergy in 1,407 school children found 11% of children reporting adverse reactions to foods, and 5% of 1,431 children showed a positive SPT reaction mostly directed against peanut and pineapple [41]. In another study, life prevalence of self-reported food allergy in Maputo, Mozambique was 19.1% with seafood (specific seafood not identified) (54.8%), meat (13.0%) and fruits and vegetables (13%) being the most frequently reported allergenic foods [42]. Other exotic foods such as the mopane worm, a high protein delicacy, consumed in some regions in Africa have been reported to cause allergic reaction in some individuals [43].

More recently, Hossny *et al.*[44] conducted a study of 100 children in Cairo (Egypt) diagnosed to have allergic diseases and found positive skin prick tests with peanut extract in seven children (7%). Specific IgE results of these children ranged from 17–24 kUA/L. The 7 children sensitized to peanut had positive family history of allergic diseases. Six of the 7 children consented to oral challenge studies and 3 were confirmed to have peanut allergy. Of the other children, 10 had confirmed allergy to other foods including egg allergy in 2, fish in 3, cow’s milk in 2, sesame in 1, banana in 1, and prunes in 1. Nine of these ten children were not sensitized to peanut, however, one of them was sensitized to both peanut and bananas [44]. In their conclusion, the authors stated that peanut allergy in Egypt is underestimated and that the sensitization rates may be even higher than previously thought.

In Morocco, 9.5% of 442 patients participating in a cross-sectional study in the Fes-Meknes region reported food allergies primarily to eggs (4.2%), peanuts (2.5%) and wheat flour (0.4%) [45]. Another study found 45% of 160 atopic Moroccan children to have sensitization to food [46]. A study from Mauritius reported seafood to be the most common allergen [47]. In Lome (Togo), 10 out of 220 out-patients at a dermatology clinic suffering from pruritus reportedly had food allergies [48]. In Rivers State, Nigeria, 28% of respondents self-reported having allergy to a variety of foods including seafood (14.7%), cereals/legumes (11.4%), vegetable oil (1.1%) and pork (1.6%) [49].

Mbugi and Chilongola [50] in a review on allergic disorders in Africa reported allergy prevalence rates in Africa of 20-30% (i.e., includes all allergic disorders) and suggested that allergy is as important an issue as other highly morbid conditions such as human immunodeficiency virus/acquired immune deficiency syndrome (HIV/AIDS), malaria and tuberculosis. They recommended that problems of allergy and allergens in Africa as compared with other disease burdens may suggest the need for a more balanced approach based on priority. As with other developing and emerging countries, further work is required with larger sample sizes to determine prevalence rates in Africa.

### Prevalence of food allergy in South America

Prevalence data of food hypersensitivity in South America is scarce. Frequency of food allergy reported at medical consultations in Monterrey, Mexico was 2.7% with 51% of patients being under 5 yr [51]. The most frequent allergenic foods were dairy products, egg, fish, shrimp, beans, soy, chili, mango, cacao, and strawberry. Major symptoms were cutaneous in 58% of patients, followed by gastrointestinal and respiratory. In a broader study on the prevalence of allergic diseases in Mexico City, allergic rhinitis was most frequently reported (42.6%) [52]. Most important risk factors for allergic diseases were family history of atopy in first and second degree relatives, early consumption of cow’s milk, early weaning with cereal, egg, beef, fish and pulses.

A prospective, longitudinal study of 279 outpatients with symptoms suggestive of respiratory allergy visiting an allergy clinic in Mexico reported prevalence of oral allergy to peach, apple, kiwi, pear and banana in 50 to 53.8% of patients [53]. In another study of 356 allergic patients who had been admitted to tertiary care the most common food allergens found were to *Amaranthus palmeri* (43.8%), chicken (9.3%) and apple/pineapple (1.2%) [54]. Other Mexican studies have reported sensitivities to fish, milk, seafood/shellfish, egg, wheat, soy, beans, orange, onion, tomato, chicken, pork, nut, peanut, rice, lettuce and strawberry [55-59].

In a study of 408 children and adolescents between the ages of 8 months and 15 years reporting with allergies to a Chilean healthcare institution, Martinez *et al.*[60] found nearly 58% to have positive skin prick test response to dust mites primarily, but also to cow’s milk, egg, beef, seafood, peanut, soy and orange. A similar Brazilian study of patients with clinical history of food allergy found the most common food allergens to be fish, egg white, cow’s milk, wheat, peanut, soy and corn [61].

In Argentina, a phone survey found approximately one million Argentines over the age of 18 (i.e., 5%) have food allergies [62,63]. Peer-reviewed data of prevalence rates is however very limited. Acknowledgement of the paucity of data on food allergies has resulted in the formation of the Platform of Food Allergens a discussion forum of various professionals to address the issue [64].

Recently Santos *et al*. [65] highlighted reports of allergic reactions to manioc or cassava (*Manihot esculenta Crantz*) in Brazil, Mozambique and Spain. Manioc is a tuber used as a source of food and starch in many countries, in particular South America, Africa and Asia. Its allergenicity is linked to the latex-fruit syndrome which is the association of latex allergy and allergy to other plant-derived foods.

### Other adverse food reactions

Glucose-6-phosphate dehydrogenase deficiency (G6PD deficiency) which affects more than 400 million people worldwide with the highest prevalence in Asia, Africa and the Mediterranean region [66] is a disease characterized by acute hemolysis (favism) induced by oxidative stress. Although it is different from food allergy, some symptoms such as weakness and abdominal pain may be similar to food sensitivity reactions. Fava bean which is grown and consumed in many parts of the world including Asia is the most common food trigger for acute hemolysis in G6PD deficiency patients.

Eosinophilic esophagitis (EE), characterized by extensive infiltration of eosinophils in the epithelium of esophagus, has recently been associated with food allergies. While the exact causes are unknown, EE is believed to be an allergic inflammatory disease with improvements observed with food elimination diets. Symptoms of EE include weight loss, vomiting, nausea and food impaction. Of 68 cases studied in a cross-sectional study on adults with refractory reflux disease in Tehran Iran, prevalence of eosinophilic esophagitis (EE) was 8.8% [67]. Subjects enrolled in the study had upper gastrointestinal tract symptoms such as heartburn, regurgitation, noncardiac chest pain and food impaction and were non-responsive to treatment (omeprazole 20 mg/day) for at least eight weeks. Further studies on the causes of EE, exact mechanisms and prevalence rates are required in developed and emerging economies as well as in developing countries.

Gluten sensitive enteropathy, is a distinct type of food hypersensitivity defined as an abnormal immunological response to gluten/gliadin which results in a diseased state typically characterised by damage of the lining of the gastrointestinal tract (villous atrophy). Although there has been much confusion in regards to its definition (i.e., allergy or intolerance) it fits into the broader definition of a cell-mediated delayed food allergy as it is an immune mediated reaction. One of the distinguishing features of gluten sensitive enteropathy is that continued consumption of gluten by sensitive individuals results in a diseased state known as celiac disease with a variety of presenting symptoms. Various studies suggest that celiac disease may be as much of a concern in developing and emerging countries as in the developed world [68-70]. Although data is limited (Table 3), prevalence rates of 0.3% [71] and 1% [72] have been reported in Northern India, and 0.9, 1.2, 1.6 and 0.8% in Turkish school children, Turkish blood donors, Syrian blood donors and Iranian children, respectively [73,74]. These prevalence rates are similar to those reported for Europeans and people of European descent (0.4 to 1.2%) [73]. Byass *et al*. [70] have estimated global totals of the number of undiagnosed celiac children to be 2.2 million (in 2010) of whom 42,000 likely die annually. The estimated number of under-5 deaths attributable to celiac disease was highest in the African region, followed by the Eastern Mediterranean region, South-East Asian region, Pan-American region, European region and Western Pacific region.

**Table 3:** Prevalence rates of celiac disease in different populations

**Population/country**	**Description**	**Prevalence**	**Reference**
Egypt	General pediatric population	1:187	69*
European ancestry	Various countries	1:85 – 1:262	73*
India	School children in Punjab, North India	1:310	71
India	Children and adults in Delhi, India	1:96	72
Iran	Children (i.e., control asymptomatic population)	1:118	73*
Israel	Adult blood donors	1:157	73*
Kuwait	Newborns over 5 yrs	1:3000 births	69*
Lebanon	Hospitalized children	1:200	69*
Tunisia	School children in Ariana, Tunisia	1:157	69*
Tunisia	Adult blood donors	1:700	69*
Turkey	School children	1:115	73*
Turkey	Adult blood donors	1:87	73*

* and references therein.

In discussions about food allergy in the global context, therefore, the broader definition of food allergy which encompasses IgE as well as non-IgE mediated food sensitivities should be considered. This is important as the health and social implications for all food related immune-mediated responses are likely to be similar and particularly because approaches to identify and control these foods along the food chain and to manage disease burdens may be identical.

## Conclusion

Although food allergy is recognized as a growing problem in the western world, there continues to be limited data on prevalence and incidence rates in many developing countries and emerging economies. Similarities between symptoms of food allergies and those of malnutrition and other childhood diseases is of particular concern as this can prevent effective diagnosis. Additionally, as the major food sources used in international food aid programs are frequently priority allergens (e.g., peanut, milk, eggs, soybean, fish, wheat), it will be increasingly important to understand and assess the interplay between food allergy and nutrition in order to protect and identify appropriate sources of foods for sensitized sub-populations especially in disadvantaged countries and communities. Identification of infants at risk will be a critical first step in this endeavor. Indeed, studies have shown that restricted diets (as is highly likely to occur with food allergy) cause malnutrition even in the developed world [75]. Management of food allergy at the global level (i.e., in both developed, developing and emerging countries) is therefore important, and requires particular consideration in the developing world where the burden of malnutrition already represents a significant challenge [76]. As previously stated by Shek and Lee [14] large, well-designed epidemiological studies are needed so that the scale of the problem can be understood, public awareness can be increased and important food allergens can be identified. With looming food and agricultural issues such as growing hunger, malnutrition and rising food prices, food allergy, may remain a low priority if a consistent effort is not maintained to improve our understanding of its health, social and economic impact at the global level.

## Competing interests

The author declares that there are no competing interests.
